# Estimated glomerular filtration rate in clinical practice: Consensus positioning of the Brazilian Society of Nephrology (SBN) and Brazilian Society of Clinical Pathology and Laboratory Medicine (SBPC/ML)

**DOI:** 10.1590/2175-8239-JBN-2023-0193en

**Published:** 2024-04-05

**Authors:** Gianna Mastroianni Kirsztajn, Geraldo Bezerra da Silva, Artur Quintiliano Bezerra da Silva, Hugo Abensur, João Egídio Romão, Marcus Gomes Bastos, Viviane Calice-Silva, Lilian Pires de Freitas do Carmo, Tainá Veras de Sandes-Freitas, Patrícia Ferreira Abreu, Bruna Dolci Andreguetto, Luiz Gustavo Ferreira Cortes, Maria Gabriela de Lucca Oliveira, Luisane Maria Falci Vieira, José A. Moura-Neto, Adagmar Andriolo

**Affiliations:** 1 Sociedade Brasileira de Nefrologia, São Paulo, SP, Brazil.; 2 Universidade Federal de São Paulo, Escola Paulista de Medicina, São Paulo, SP, Brazil.; 3 Universidade de Fortaleza, Centro de Ciências da Saúde, Programas de Pós-Graduação em Ciências Médicas e Saúde Coletiva, Fortaleza, CE, Brazil.; 4 Universidade Federal do Rio Grande do Norte, Departamento de Medicina Integrada, Natal, RN, Brazil.; 5 Universidade de São Paulo, Faculdade de Medicina, São Paulo, SP, Brazil.; 6 Faculdade de Ciências Médicas e da Saúde de Juiz de Fora, Juiz de Fora, MG, Brazil.; 7 Faculdade Ubaense Ozanam Coelho, Ubá, MG, Brazil.; 8 Universidade da Região de Joinville, Joinville, SC, Brazil.; 9 Fundação Pró-Rim, Joinville, SC, Brazil.; 10 Universidade Federal de Minas Gerais, Faculdade de Medicina, Belo Horizonte, MG, Brazil.; 11 Universidade Federal do Ceará, Faculdade de Medicina, Fortaleza, CE, Brazil.; 12 Sociedade Brasileira de Patologia Clínica e Medicina Laboratorial, Rio de Janeiro, RJ, Brazil.; 13 Dasa – Diagnósticos da América S.A., São Paulo, SP, Brazil.; 14 Escola Bahiana de Medicina e Saúde Pública, Salvador, BA, Brazil.; 15 Hospital Israelita Albert Einstein, Laboratório Clínico, São Paulo, SP, Brazil.

**Keywords:** Kidney Function Tests, Creatinine, Glomerular Filtration Rate, Diagnostic Tests, Cystatin C, Renal Insufficiency, Chronic, Race Factors

## Abstract

Chronic kidney disease (CKD) represents one of today’s main public health problems. Serum creatinine measurement and estimation of the glomerular filtration rate (GFR) are the main tools for evaluating renal function. There are several equations to estimate GFR, and CKD-EPI equation (Chronic Kidney Disease – Epidemiology) is the most recommended one. There are still some controversies regarding serum creatinine measurement and GFR estimation, since several factors can interfere in this process. An important recent change was the removal of the correction for race from the equations for estimating GFR, which overestimated kidney function, and consequently delayed the implementation of treatments such as dialysis and kidney transplantation. In this consensus document from the Brazilian Societies of Nephrology and Clinical Pathology and Laboratory Medicine, the main concepts related to the assessment of renal function are reviewed, as well as possible existing controversies and recommendations for estimating GFR in clinical practice.

## Introduction

Nowadays, it is well established that chronic kidney disease (CKD) has a high prevalence and a significant economic cost, especially when renal replacement therapy becomes necessary. Furthermore, CKD is an important cardiovascular risk factor. Due to these characteristics, CKD has become a major public health issue^1^.

Individuals with a glomerular filtration rate (GFR) < 60 mL/min/1.73 m^2^, or changes in a kidney injury biomarker (e.g. albuminuria) or with structural/anatomical changes, even with a GFR equal to or greater than 60 mL/min/1.73 m^2^, for a period longer than three months, are considered to have CKD^2^.

In clinical practice, GFR may be assessed simply from serum creatinine, the concentration of which is inversely related to GFR. Serum (or plasma) creatinine presents some limitations, such as kidney function testing, being affected by non-renal, pre-analytical and analytical factors. However, for most clinical purposes, the accuracy of GFR assessment methods based on the administration of exogenous substances (e.g. inulin, iohexol, Tc^99m^-diethylenetriamine pentaacetic acid, Cr^51^-ethylenediamine tetraacetic acid) is replaced by the practicality of estimating GFR with serum creatinine.

Through the estimation of GFR obtained using previously validated formulas, it is possible to assess the presence of CKD and its current stage. This enables the establishment of a treatment plan aimed at preventing the disease from progressing to more advanced stages. In addition, through estimated GFR (eGFR), it is possible to identify which patients require follow-up with a specialist and those who can remain under follow-up with the primary care team.

## Laboratory Assessment of Kidney Function

### Measurement of Serum Creatinine

Clinical assessment of kidney function is part of routine medical care, with serum creatinine being the most widely used laboratory test to evaluate it. In an assessment in Canada, creatinine was the second most requested laboratory test for outpatients and the third for inpatients and emergency cases^3^.

As creatinine is predominantly eliminated by glomerular filtration, with only a minimal fraction being secreted by tubular cells, serum creatinine concentration has an inverse association with renal function. An elevation corresponding to a two-fold increase in serum creatinine concentration means an approximate 50% reduction in GFR.

Creatinine is the product of the metabolism and degradation of creatine and phosphocreatine in skeletal muscle cells, and it is generated at a relatively constant rate and consequently released steadily into the bloodstream. Given its low molecular weight, it is widely diffused through all cell membranes, being present in blood plasma, other biological fluids and inside erythrocytes.

Creatinine production is largely dependent on lean body mass and some other individual characteristics, such as age, sex, dietary habits and physical activity. Regarding these characteristics, we would highlight age extremes (in pediatric and geriatric groups), the intake or not of animal proteins (reduced or absent in vegetarians and vegans), and muscle mass extremes (malnutrition, sarcopenia). Special cases are worth considering, such as people who have had limbs amputated or those in advanced stages of cachexia. Creatinine serum levels may be above the individual’s baseline values a few hours after a meal with a high content of animal proteins^4^.

The creatinine excretion pathway is predominantly by glomerular filtration; however, under normal conditions, around 7% to 10% of urinary creatinine originates from tubular secretion^5^.

In whole blood, creatinine is stable for 24 hours; in serum, stability lasts up to 7 days at room temperature (20–25°C) or refrigerated between 2 and 8°C. In serum samples frozen at minus 20°C, it is stable for up to 3 months, even with freeze-thaw cycles^6^.

The available methods for measuring serum and urine creatinine include the colorimetric method based on Jaffe’s reaction and the enzymatic one, both potentially traceable by Isotope Dilution Mass Spectrometry (ID-MS), and eligible for calibration using the National Institute of Standards and Technology Standards Reference Material (NIST SRM). Enzymatic assays are less influenced by non-creatinine chromogens compared to colorimetric assays^7,8^, showing lower variability and thus being more suitable for routine laboratory use. However, they are more expensive and have not been adapted for all automated analytics platforms available on the Brazilian market^9,10^.

Traceable creatinine is referred to as the creatinine measured by a diagnostic kit, which is traceable to international reference standards determined by ID-MS. Creatinine is determined in human reference materials by the National Institute of Standards and Technology using ID-MS, which provides the standards to diagnostic kit manufacturers, so they can calibrate their assays^11^.

Some endogenous substances, depending on their concentration, could significantly interfere with the results obtained by Jaffe’s reaction-based method: bilirubin, glucose, proteins, pyruvate, β-hydroxybutyric acid, in addition to many drugs such as cephalosporins, dobutamine, lidocaine^12^. Hemolyzed samples or those containing fetal hemoglobin above the reference ranges may yield negative creatinine results with Jaffe’s reaction. Lipemic samples may yield inaccurate results, regardless of the method used^12^.

Since urine generally contains little or no protein compared to serum, and no chromogens, both the enzymatic and Jaffe methods are suitable for measuring creatinine in this matrix^13^.

It is important to note that the influence of interfering substances is greater at low creatinine concentrations, i.e. within the reference range, than at higher concentrations, and that calibration standardization does not minimize analytical interferences^13^.

In general, children have lower serum creatinine concentrations than adults, thus having their own reference intervals. Although there may be differences between populations, the limits defined by the Canadian Laboratory Initiative in Pediatric Reference Intervals (CALIPER)^13^ study should guide laboratories and pediatricians in the most accurate interpretation of test results. There are some transfer studies to other analytical platforms and local populations, as recommended by the Clinical and Laboratory Standards Institute (CLSI)^14,15^. Another source of reference intervals for serum creatinine in children is the Pediatric Reference Range Initiatives database from the International Federation for Clinical Chemistry and Laboratory Medicine (IFCC)^16^.

### Creatinine Clearance

It is not recommended to consider the reference value limits of serum creatinine as an index for assessing kidney function, with estimated GFR being considered more sensitive and specific as a biomarker of renal function^17^.

GFR is measured by the clearance of a substance freely filtered by the glomeruli, ideally without tubular reabsorption or secretion and without being metabolized or eliminated by extrarenal pathways, expressed in mL/min/1.73 m^2^. Clearance is calculated according to the formula (UV/P) × (1.73/A), where U = urinary concentration of the substance; V = urinary volume per unit of time, usually per minute; P = plasma concentration of the substance; and A = patient’s body surface area. The (1.73/A) section of the formula allows the result to be normalized to a standard body surface area of 1.73 m^2^. Serum and urine concentrations should be expressed in the same units, the urinary volume expressed in mL and the collection time, in minutes, should be measured with great precision^17^.

GFR is measured more precisely using exogenous substances such as inulin, some radioisotopes like Cr^51^-ethylenediaminetetraacetic acid (Cr^51^-EDTA), I^125^-iothalamate, Tc^99m^-diethylenetriamine pentaacetic acid (Tc^99m^-DTPA) or radiographic contrast media such as iohexol. These procedures require some substance to be injected into the patient, are technically complex, demand special equipment, are time-consuming, expensive and have potential side effects. For these reasons, in clinical practice, endogenous biomarkers have been used to measure or estimate GFR, aiming to investigate CKD, in particular serum creatinine and cystatin C^18^.

The time of urine collection should allow for an evaluation with the lowest degree of spot variations, usually 24 hours or 12 hours. However, these extended collections introduce possible sources of error, whether in measuring urine volume or in setting the time. Therefore, shorter periods could be used, aiming for accuracy in timing urine collection and the complete emptying of the bladder before the first and last urine collections. Another critical condition is the patient’s hydration status during the test. The recommended practice is for the patient to receive a fluid load of 20 mL/kg body weight of water when starting the test, followed by replacement equivalent to the urinary volume produced in each period. The diagnostic usefulness of creatinine clearance largely depends on adherence to the standardized protocol for collecting blood and urine samples^19^.

When glomerular filtration is within the reference ranges, creatinine clearance is practically overlapping with that of inulin. Results 7% to 10% higher are observed as consequence of tubular secretion, but as the kidney disease progresses to more advanced stages, tubular secretion of creatinine increases, leading to overestimation of GFR by serum creatinine clearance. Additionally, there is an increase in the extrarenal clearance of creatinine, with an increase in intestinal bacteria with creatininase activity^5^.

Some drugs work directly on renal tubular cells, influencing their ability to secrete creatinine, thus altering the ratio between filtered and secreted creatinine. Among the drugs that reduce tubular secretion are salicylates, cimetidine, trimethoprim, quinine, procainamide and several others. Among the drugs that increase tubular secretion are triamterene, spironolactone, and amiloride^5^.

The individual circadian variation may be up to 25%, and the reference ranges for adult subjects are: men, 85 to 125 mL/min; and women, 75 to 115 mL/min. GFR physiologically decreases with aging by approximately 1 mL/min/year of age^20,21^. During pregnancy, due to the significant increase in renal blood flow, creatinine clearance increases by 50% or even more^22^.

Another important concept is renal functional reserve, characterized as the capacity of the kidneys to increase GFR in response to physiological or pathological stimuli^23^. The best-known and most common stimulus that boosts this reserve, increasing GFR, is protein overload. Renal functional reserve is calculated by the difference between stimulated GFR (increased by the stimulus) and baseline GFR. Some practical examples are described as follows: vegetarian individuals have a lower baseline GFR compared to the general population, and higher renal functional reserve; patients with chronic nephropathies and a solitary kidney usually have reduced or absent renal functional reserve, even if they have normal GFR^23^.

As the primary measure of renal function used in current clinical practice, serum creatinine measurement and estimation of GFR are recommended. It is not advised to use reference values for normal serum creatinine to guide the interpretation of laboratory test results, and the eGFR should be reported in every serum creatinine test^5^.

### Measurement of Serum Cystatin C

Unlike creatinine, serum cystatin C concentration is less dependent on age, sex, ethnicity, diet and muscle mass^24,25^. There are references to alterations in its concentration in patients taking corticosteroids^26^, with neoplastic processes^27^ and with thyroid disease^28^.

Cystatin C is a low molecular weight protein produced continuously and constantly by practically all nucleated cells in the human body, being removed from the bloodstream exclusively by glomerular filtration. After filtration, it is reabsorbed and metabolized without undergoing secretion in the proximal tubule^29^. Consequently, although cystatin C is freely filtered by the glomeruli, its urinary clearance cannot be assessed^29^.

Cystatin C can be measured in serum by immunoassays, such as particle-enhanced nephelometric immunoassay (PENIA) or particle-enhanced turbidimetric immunoassay (PETIA)^30^. Just like creatinine, cystatin C has also become traceable through the ERM-DA471/IFCC^31^ reference material, and equations for estimating GFR should use cystatin C values obtained by methods validated by the IFCC^32^.

However, measuring cystatin C is more expensive and not always as easy and accessible in many laboratories as creatinine measurement. It is best used for confirming results or in cases where creatinine measurement may suffer from pre-analytical interference. The reference ranges described differ little with regard to sex and age. For women, the reference range is reported as 0.52 to 0.90 mg/L, with an average of 0.71 mg/L, and for men, 0.56 to 0.98 mg/L, with an average of 0.77 mg/L^30^. Similarly to creatinine, values decrease from birth until the first year of life, remaining relatively stable in adulthood^33,34^.

Quantifying its serum level is more sensitive than that of creatinine for the assessment of renal glomerular filtration function^35,36^, and it is a better biomarker in the early detection of renal function decline, both in adults and children. The UK’s National Institute for Health and Care Excellence (NICE) guidelines concluded that estimating GFR with cystatin C is more specific for predicting important disease outcomes than using creatinine^37^.

## Considerations on Equations for Estimating Glomerular Filtration Rate

### Equations for Use in Adults

The first studies conducted to estimate kidney function using creatinine-based formulas date back to the middle of the 20th century^38,39^. One of the best-known equations for estimating GFR was developed by Donald W. Cockcroft and M. Henry Gault in 1976^40^. The authors used data from 249 men with creatinine clearance between 30 and 130 mL/min. The parameters considered were age, sex, body weight and creatinine concentration. This equation was not parameterized for standard body surface area and was not developed with creatinine standardized by ID-MS. In addition, the formula was developed considering 24-hour urine creatinine clearance as the reference standard, making it an estimate of creatinine clearance rather than GFR precisely^40^. Therefore, its use is not recommended for clinical purposes, being restricted to research. The only current advantage of this formula is that it could be manually calculated, without the need for applications.

In 1999, the Modification of Diet in Renal Disease (MDRD) study presented an equation^41^ developed from a cohort of 983 men and 645 women, predominantly white adults, with different levels of renal disease, enrolled in a study aimed at evaluating the effect of low-protein diets on the progression of CKD. Originally, this formula included six parameters: age, sex, race, urea nitrogen, albumin and serum creatinine. It was later simplified to just four variables: age, sex, race and serum creatinine, and was identified as 4-MDRD^42^. In both equations, there is a specific factor designed to discriminate between black individuals and a correction factor for the standard body surface area of 1.73 m^2^. Given the inaccuracy of estimation at high GFR levels, the National Kidney Disease Education Program (NKDEP) recommends that when eGFR is above 60 mL/min/1.73 m^2^, the value should only be reported as exceeding this limit.

A second modification to this formula occurred in 2009, when the measurement of serum creatinine using assays with calibration traceable to international reference materials measured by mass spectrometry and isotope dilution (MS-ID) methodology was introduced^43^. To derive this equation, we used data from 1,628 patients with CKD at different stages, all participating in the same MDRD study. GFR was measured directly with iothalamate-I^125^. This equation has been shown to have greater accuracy and precision when compared to the previous 4-MDRD equation, especially for GFR above 60 mL/min/1.73 m^2^. A separate discriminatory coefficient was maintained for black patients^43^. This equation has been widely used, including in Brazil, where the racial criterion is less pronounced.

In 2009, a new equation named CKD-EPI (Chronic Kidney Disease – Epidemiology) was developed, with greater accuracy than MDRD for estimating GFR^44^. In 2022, the Kidney Disease: Improving Global Outcomes (KDIGO) developed a new version of the formula for estimating GFR using serum creatinine as a biomarker and excluding any race-related correction factors^45^. This equation was developed based on data from two meta-analyses. One included 10 studies with 8,254 participants, of whom 31.5% were black, using serum creatinine measurement. The other included 13 studies with 5,352 participants, of whom 39.7% were black, also using serum creatinine measurement.

The Scientific Societies recommend replacing previously used equations with equations that do not discriminate patients based on race criteria and use only creatinine values or the association of creatinine and cystatin C. Validation studies already available demonstrate that these equations are sufficiently accurate and inclusive^46^.

It is reasonable to believe that the new equation proves to be more appropriate for the Brazilian population, since its formulation considered the average of the differences observed among participants of each race. The immediate effect of applying the equation without racial discrimination will not significantly affect non-black patients, but it will increase the chance of diagnosing CKD in black people, enabling earlier treatment to be provided.

It is worth remembering that the new equations, without race criteria, were also developed based on data obtained from adult subjects, in outpatient care, without significant comorbidities. Thus, the limitations of use described for the previous equations remain valid.

Equations for adults

Cockcroft & Gault (1976)

eGFR (mL/min) = [(140 – age) × weight × (0.85 if female)]/72 x creatinine.

4-MDRD

eGFR (mL/min/1.73 m^2^) = 175 × creatinine^–1.154^ × age^–0.203^ × 1.212 (if black) × 0.742 (if female).

CKD-EPI (2009)

eGFR (mL/min/1.73 m^2^) = 141 min (creatinine/k, 1)^α^ max (creatinine/k, 1)^–1.209^ × 0.993^age^ × 1.018 (if female) × 1.159 (if black).

k is 0.7 for women and 0.9 for men; α is 0.329 for women and 0.411 for men; min is the minimum creatinine divided by k or by 1; and max is the maximum creatinine divided by k or by 1.

CKD-EPI (2012) using only cystatin C

eGFR (mL/min/1.73 m^2^) = 133 × min (cistatine/0.8, 1)^–0.499^ × max (cistatine/0.8, 1)^–1.328^ × 0.996^age^ × 0.932 (if female).

CKD-EPI (2012) using creatinine and cystatin

eGFR (mL/min/1.73 m^2^) = 135 min (creatinine/k, 1)^α^ × max (creatinine/k, 1)^0.601^ × min (cistatine/0.8, 1)^0.375^ × max (cistatine/0.8, 1)^0.711^ 0.995^age^ × 0.969 (if female) × (1.08 if black)

k is 0.7 for women and 0.9 for men; α is 0.248 for women and 0.207 for men.

CKD-EPI (2021) using only creatinine

eGFR (mL/min/1.73 m^2^) = 142 x min (creatinine/k, 1)^α^ × max (creatinine/k, 1)^1.200^ × 0.9938^age^ × 1.012 (if female).

k is 0.7 for women and 0.9 for men; α is 0.241 for women and 0.302 for men.

CKD-EPI 2021 using creatinine and cystatin C

eGFR (mL/min/1.73 m^2^) = 135 × min (creatinine/κ, 1)^α^ × max (creatinine/κ, 1)^−1.200^ × min (cistatine/0.8, 1)^−0.323^ × max (cistatine/ 0.8, 1)^−0.778^ × 0.9961^age^ × 0.963 (if female).

k is 0.7 for women and 0.9 for men; α = −0,219 (for women) and −0.144 (for men); min is the minimum creatinine divided by k or by 1; and max is the maximum creatinine divided by k or by 1.

Note: in all equations, age is expressed in years, serum creatinine in mg/dL, cystatin C in mg/L, body weight in kilograms.

### Equations for Use in Pediatrics

Currently, there are more than 10 equations to estimate GFR in the pediatric population, and their application is recommended for children over two years of age^2^. Similarly to the equations designed for adults, some of them are based exclusively on serum creatinine; others on cystatin C; and still others on a combination of these two biomarkers.

One of the equations for estimating GFR in children was described by Schwartz et al. in 1976, and it relates a constant, the child’s body height, and the serum creatinine concentration^47^. Using statistical analysis of data from 186 children, the authors derived a formula that allows GFR to be estimated, including parameterization for a standard body surface area of 1.73 m^2^. When validated in a group of 223 children, the equation proved to be quite consistent with directly measured clearance, either by creatinine or by inulin clearance.

In the same year, Counahan et al. published another equation, using a single constant and the child’s height-to-serum creatinine ratio^48^.

In 1987, Schwartz et al. published a modification to the original equation, in which the constant value was related to the child’s age. The remaining parameters were maintained^49^.

The equations currently considered the most appropriate for estimating GFR in children are those developed and validated by the Chronic Kidney Disease in Children (CKiD) study, published in 2009 and 2012, known as the CKiD Schwartz equations. They relate serum creatinine alone or in combination with cystatin C, blood urea nitrogen (BUN), body height and the child’s biological sex.

They were developed from data on children with CKD, using an enzymatic method for measuring creatinine, traceable by isotope dilution mass spectrometry (IDMS). These equations proved to be highly accurate, especially in the range between 15 and 75 mL/min/1.73 m^2^. According to the authors, values above 75 mL/min/1.73 m^2^ should only be reported on this basis, and not as a specific number^50,51^.

Despite the accuracy of these equations, there is the inconvenience of needing to know the values of constants, such as body height, which are not always available when the formulas are applied. Due to this, and the particularities and limitations of creatinine measurement, estimations of eGFR in the pediatric population have been formulated using cystatin C. Among these, the Schwartz formula is particularly noteworthy^51^.

## Evidence on the Use of Estimated GFR From Serum Creatinine

The CKD-EPI equation is the one recommended for estimating GFR in adults^43^. Regression models were used in the development of the equations, using serum creatinine and demographic data.

In clinical conditions involving muscle wasting or in amputee patients, the use of creatinine-based eGFR is limited. Other situations not related to glomerular filtration that influence blood creatinine levels and, consequently, eGFR are: 1. Biological sex: men generally have higher creatinine levels than women for the same level of glomerular filtration; 2. Ethnicity: creatinine levels are higher in Afro-Caribbean individuals for the same level of eGFR; 3. Recent dietary intake: cooked meat and fish contain creatine, which is rapidly absorbed; 4. Drugs (*in vivo* effect): for example, cimetidine and trimethoprim block tubular secretion of creatinine. Typically, 7–10% of creatinine excretion is tubular, a percentage that increases with the loss of glomerular filtration; 5. Extrarenal creatinine clearance: it becomes more significant in CKD patients due to degradation, resulting from bacterial overgrowth in the small intestine; 6. Serum creatinine concentration remains within the reference range until a reduction in GFR of approximately 50% in non-elderly individuals and around 70% in the elderly occurs; and 7. Use of eGFR in acute kidney injury: GFR does not decrease linearly with the increase in serum creatinine, thereby restricting its applicability in clinical contexts of acute decline in glomerular filtration^52^.

Additionally, some analytical issues should be observed when using eGFR from creatinine: 1. Specificity: pseudochromogens (e.g. ketones, ascorbic acid, pyruvate, guanidine, cephalosporins, proteins) may result in false-positive reactions when using Jaffe’s colorimetric method; 2. Drugs (analytical effect): for example, metamizole causes a false increase in creatinine when using Jaffe’s colorimetric method, while phenindione and dipyrone cause a decrease in creatinine when some enzymatic methods are used; 3. Spectral interferences, such as jaundice, lipemia, and hemolysis, can yield false, elevated or reduced creatinine results, depending on the assay conditions; and 4. Methodological variation: results may differ between laboratories^53^.

## Correction by Race in Equations for Estimating GFR: Current Recommendation

The CKD-EPI equation, recommended for estimating GFR when accounting for race, may overestimate the GFR, leading to underdiagnosis of CKD. This finding was verified within the Black population in the United States and is possibly related to the fact that race is a social concept that does not necessarily correlate with underlying genotypic or metabolic differences among individuals. The racial parameters in eGFR calculation do not account for people who self-identify as multiracial, who refuse to answer questions about their race or are assigned an improper definition of race based on arbitrary phenotypic traits such as skin tone, hair type or even social status. In addition to being inappropriate for GFR calculation, the “race” parameter has been questioned in several algorithms in clinical practice^53,54^. The very existence of potential biological differences among distinct “human races” is arbitrary, since it dates back to the 18th century to justify European expansionism and the supposed superiority of the white race^52^. This notion lacks relevance in medical practice.

Considering the widespread use of eGFR in nephrology practice, this inaccuracy resulting from the inclusion of “race” factor in the equation may affect several aspects of patient care. Particularly in black patients, this may affect the way drugs are administered or even proscribed, the speed at which patients are diagnosed and referred for nephrology consultation for CKD treatment, eligibility for clinical trials and access to kidney transplant waiting lists.

In 2021, a new CKD-EPI equation without the use of race correction was proposed^55^. Even in the absence of this correction, equations for estimating GFR, including the CKD-EPI, have been developed and validated mostly for the North American population^55^, with their accuracy for other populations, such as the Brazilian one, still poorly studied^56^.

### Estimated GFR From Cystatin C

Cystatin C is a low molecular weight plasma protein (12.8 kDa) that is freely filtered by the glomerulus. Considering that it is completely eliminated from the circulation only through this pathway, it has the potential to be used as a GFR biomarker^56^. Cystatin C is an endogenous cysteine protease inhibitor synthesized by all nucleated cells and has a strong correlation with eGFR. Its production is relatively independent of sex, muscle mass, dietary influences and nychthemeral cycles, and may be influenced by inflammation, neoplastic processes, thyroid dysfunction and smoking^57^.

Equations for estimating GFR based on cystatin C^58^ have also been developed, including equations that incorporate both cystatin C and creatinine^59^. Despite showing only modest improvements in eGFR accuracy compared to creatinine-based equations, there is growing evidence that the use of cystatin C outperforms creatinine in risk stratification, especially regarding cardiovascular risk. Furthermore, cystatin C-based eGFR is less impacted by age, dietary intake and race, and may be beneficial in cases where the correlation between muscle mass, creatinine and GFR is compromised (e.g. consumptive diseases and in amputee patients).

The KDIGO^2^ Guidelines provide evidence-based recommendations for determining eGFR, as outlined below:

- Serum creatinine or its clearance is recommended to obtain the eGFR for initial investigation (1A);
- Additional laboratory tests, such as cystatin C or its eGFR are suggested as confirmatory exams in specific circumstances when GFR based on serum creatinine is less reliable (2B);
- It is recommended that clinicians use the equation for estimating GFR derived from serum creatinine (eGFR) rather than relying on the absolute value of serum creatinine (1B).

### Online Calculators

Numerous online calculators are available for solving equations that estimate GFR, greatly facilitating their application in the routine of both clinicians and specialists. Similarly, with the introduction of computerized systems in clinical laboratories, it has become common for the estimate to be provided as a complement to the request for serum creatinine measurement. Clearly, this practice is still consolidating in our setting, and it is restricted to equations that use only creatinine. The introduction of equations based only on cystatin C or in combination with creatinine will pose operational challenges, since the measurement of these two parameters will require an explanation from the doctor.

Below, some websites that provide calculators.

https://sbn.org.br/medicos/utilidades/calculadoras-nefrologicas/ckdepi

https://www.kidney.org/professionals/Kdoqi/gfr_calculatorPed

https://www.mdcalc.com/calc/43/creatinine-clearance-cockcroft-gault-equation

https://www.mdcalc.com/calc/3939/ckd-epi-equations-glomerular-filtration-rate-gfr

https://www.mdcalc.com/calc/10008/revised-schwartz-equation-glomerular-filtration-rate-gfr-2009

## Recommendations for Reports

The current recommendations regarding laboratory test reports are that:

- Serum creatinine concentration is reported in mg/dL and its value should be rounded to the nearest hundredth of a whole number, meaning it should be a number with two decimal places.
- Estimated GFR is included in the creatinine result report and its value is reported and rounded to the nearest whole number. It should be related to the body surface area of 1.73 m^2^ in adults, using the unit mL/min/1.73 m^2^.
- Creatinine-based eGFR value below 60 mL/min/1.73 m^2^ is reported as “decreased”.
- The value of serum cystatin C concentration should be reported rounded to the nearest hundredth of a whole number when expressed in conventional units (mg/L).
- eGFR values based on equations using only cystatin C or in combination with creatinine should be reported rounded to the nearest whole number and in relation to body surface area, using the unit mL/min/1.73 m^2^.
- eGFR levels based on equations using only cystatin C or in combination with creatinine, below 60 mL/min/1,73 m^2^, are reported as “decreased”.
- The equations used are clearly reported, including their references.

## Situations in which the Use of Estimated GFR is not Recommended

The GFR estimates were developed under situations of stable serum creatinine. Its serum level is known to depend on multiple factors, including muscle mass and activity, dietary intake, tubular secretion and elimination through the gastrointestinal tract. These factors are not considered determinants of glomerular filtration.

In situations where creatinine concentration is unstable, such as acute kidney injury, eGFR errors are more pronounced.

Proper use and interpretation of eGFR requires its evaluation within the patient’s clinical context. Extremes of age, such as children and the elderly, have particularities that should be considered.

## Use of Estimated GFR as a Tool for Prevention/Early Detection of CKD

Studies on early diagnosis of CKD suggest that essential laboratory tests for initial diagnosis should include the measurement of proteinuria. It is noteworthy that KDIGO recommends determining the albumin/creatinine ratio in a spot urine sample, and also determining serum creatinine concentration, sensitized by the associated determination of estimated GFR. The estimation enables early diagnosis of a greater number of cases involving kidney failure.

CKD screening through eGFR and albuminuria measurement has been recommended for all patients with risk factors for CKD, such as hypertensive and diabetic patients, for example. Individuals with risk factors should be assessed annually, with recommendations for reassessment of eGFR parameters and urine albumin-creatinine ratio (uACR) every six months, according to the clinical context and results or their variation over the course of monitoring. Currently, it is recommended to test for albuminuria using a spot urine sample (urine albumin/creatinine ratio).

Patients with stages G4 and G5 CKD should be referred to a nephrologist for follow-up and appropriate management of disease complications^44^.

## Conclusion

Although still underutilized, proteinuria or albuminuria levels and eGFR are valuable resources in the detection and staging of CKD, as well as in predicting the risk of CKD progression and CVD. In this manuscript, we exclusively concentrated on assessing GFR, while acknowledging the significance and sensitivity of proteinuria and albuminuria as crucial biomarkers of kidney damage.

In accordance with the guidelines of the American Association for Clinical Chemistry (AACC) and the National Kidney Foundation (NKF), the Brazilian Society of Clinical Pathology/Laboratory Medicine (SBPC/ML) and the Brazilian Society of Nephrology (SBN), the preferred equations for estimating GFR in adult individuals are as follows:

- CKD-EPI (2021), using only serum creatinine;
- CKD-EPI (2021), using serum creatinine and cystatin and
- CKD-EPI (2012), using only serum cystatin C.

For the pediatric population, the equations recommended by NKF, SBN and SBPC/ML are:

- Schwartz equation (2009), based on serum creatinine;
- CKiD Schwartz equation (2012), based on serum cystatin C; and
- CKiD Schwartz equation (2012), based on serum creatinine and cystatin C.
