# Erratum: Estimated glomerular filtration rate in clinical practice: consensus positioning of the Brazilian Society of Nephrology (SBN) and Brazilian Society of Clinical Pathology and Laboratory Medicine (SBPC/ML)

**DOI:** 10.1590/2175-8239-JBN-2023-0193eren

**Published:** 2025-04-14

In the article “Estimated glomerular filtration rate in clinical practice: consensus positioning of the Brazilian Society of Nephrology (SBN) and Brazilian Society of Clinical Pathology and Laboratory Medicine (SBPC/ML)”, with DOI code number: https://doi.org/10.1590/2175-8239-JBN-2023-0193en, published in the journal Brazilian Journal of Nephrology, 46(3):e20230193, 2024, in the page 6:

**Where it was written:**

CKD-EPI (2021) using only creatinine

eGFR (mL/min/1.73 m^2^) = 142 × min (creatinine/k, 1)^α^ × max (creatinine/k, 1)^1.200^ × 0.9938^age^ × 1.012 (if female).

k is 0.7 for women and 0.9 for men; α is 0.241 for women and 0.302 for men.

**Should read:**

CKD-EPI (2021) using only creatinine

eGFR (mL/min/1.73 m^2^) = 142 × min (creatinine/k, 1)^α^ × max (creatinine/k, 1)^-1.200^ × 0.9938^age^ × 1.012 (if female).

k is 0.7 for women and 0.9 for men; α is 0.241 for women and 0.302 for men.

