# Supplementary material for: Estimated glomerular filtration rate in clinical practice: Consensus positioning of the Brazilian Society of Nephrology (SBN) and Brazilian Society of Clinical Pathology and Laboratory Medicine (SBPC/ML)
Source: J Bras Nefrol. 2024 Apr 5;46(3):e20230193. doi: 10.1590/2175-8239-JBN-2023-0193en (PMC11300030; doi:10.1590/2175-8239-JBN-2023-0193en)
Supplement: Translation

# Translation: Estimativa da taxa de filtração glomerular na prática clínica: posicionamento consensual da Sociedade Brasileira de Nefrologia (SBN) e Sociedade Brasileira de Patologia Clínica e Medicina Laboratorial (SBPC/ML)

**DOI:** 10.1590/2175-8239-JBN-2023-0193pt

**Authors:** Gianna Mastroianni Kirsztajn, Geraldo Bezerra da Silva, Artur Quintiliano Bezerra da Silva, Hugo Abensur, João Egídio Romão, Marcus Gomes Bastos, Viviane Calice-Silva, Lilian Pires de Freitas do Carmo, Tainá Veras de Sandes-Freitas, Patrícia Ferreira Abreu, Bruna Dolci Andreguetto, Luiz Gustavo Ferreira Cortes, Maria Gabriela de Lucca Oliveira, Luisane Maria Falci Vieira, José A. Moura-Neto, Adagmar Andriolo

**Affiliations:** 1 Sociedade Brasileira de Nefrologia, São Paulo, SP, Brasil.; 2 Universidade Federal de São Paulo, Escola Paulista de Medicina, São Paulo, SP, Brasil.; 3 Universidade de Fortaleza, Centro de Ciências da Saúde, Programas de Pós-Graduação em Ciências Médicas e Saúde Coletiva, Fortaleza, CE, Brasil.; 4 Universidade Federal do Rio Grande do Norte, Departamento de Medicina Integrada, Natal, RN, Brasil.; 5 Universidade de São Paulo, Faculdade de Medicina, São Paulo, SP, Brasil.; 6 Faculdade de Ciências Médicas e da Saúde de Juiz de Fora, Juiz de Fora, MG, Brasil.; 7 Faculdade Ubaense Ozanam Coelho, Ubá, MG, Brasil.; 8 Universidade da Região de Joinville, Joinville, SC, Brasil.; 9 Fundação Pró-Rim, Joinville, SC, Brasil.; 10 Universidade Federal de Minas Gerais, Faculdade de Medicina, Belo Horizonte, MG, Brasil.; 11 Universidade Federal do Ceará, Faculdade de Medicina, Fortaleza, CE, Brasil.; 12 Sociedade Brasileira de Patologia Clínica e Medicina Laboratorial, Rio de Janeiro, RJ, Brasil.; 13 Dasa – Diagnósticos da América S.A., São Paulo, SP, Brasil.; 14 Escola Bahiana de Medicina e Saúde Pública, Salvador, BA, Brasil.; 15 Hospital Israelita Albert Einstein, Laboratório Clínico, São Paulo, SP, Brasil.

**Keywords:** Testes de Função Renal, Creatinina, Taxa de Filtração Glomerular, Testes diagnósticos, Cistatina C, Insuficiência Renal Crônica, Fatores Raciais

## Abstract

A doença renal crônica (DRC) representa um dos principais problemas de saúde pública da atualidade. A dosagem da creatinina sérica e a estimativa da taxa de filtração glomerular (TFG) são as principais ferramentas para avaliação da função renal. Para a estimativa da TFG, existem diversas equações, sendo a mais recomendada a CKD-EPI (*Chronic Kidney Disease – Epidemiology*). Existem ainda algumas controvérsias com relação à dosagem da creatinina sérica e da estimativa da TFG, uma vez que vários fatores podem interferir nesse processo. Uma importante mudança recente foi a retirada da correção por raça das equações para estimativa da TFG, que superestimavam a função renal, e consequentemente retardavam a implementação de tratamentos como diálise e transplante renal. Neste documento de consenso da Sociedade Brasileira de Nefrologia e Sociedade Brasileira de Patologia Clínica e Medicina Laboratorial são revisados os principais conceitos relacionados à avaliação da função renal, possíveis controvérsias existentes e recomendações para a estimativa da TFG na prática clínica.

## Introdução

Nos nossos dias, está bem estabelecido que a doença renal crônica (DRC) tem alta prevalência e um elevado custo econômico, particularmente quando há necessidade de terapia renal substitutiva. Além disso, a DRC é um importante fator de risco cardiovascular. Em função dessas características, a DRC tornou-se um relevante problema de saúde pública^1^.

Considera-se que tem DRC todo indivíduo com taxa de filtração glomerular (TFG) < 60 mL/min/1,73 m^2^, ou com alterações em um marcador de lesão renal (por exemplo, albuminúria) ou com alterações estruturais/anatômicas, mesmo com TFG igual ou maior que 60 mL/min/1,73 m^2^, por um período superior a três meses^2^.

Na prática clínica, a TFG pode ser avaliada, de maneira simples, a partir da creatinina sérica, cuja concentração guarda relação inversa com a TFG. A creatinina sérica (ou plasmática) apresenta algumas limitações, como teste de função renal, sendo afetada por fatores não renais, pré-analíticos e analíticos. Entretanto, para a maioria dos fins clínicos, a acurácia dos métodos de avaliação da TFG baseados na administração de substâncias exógenas (por exemplo, inulina, iohexol, Tc^99m^-ácido dietilenotriamino pentacético, Cr^51^-ácido etilenodiamino tetra-acético) é substituída pela praticidade da estimativa da TFG com a creatinina sérica.

Através da estimativa da TFG obtida pelo uso de fórmulas previamente validadas, é possível avaliar a presença de DRC e seu estágio atual, permitindo que se estabeleça um plano de tratamento com o objetivo de prevenir a progressão da doença para estágios mais avançados. Ainda, através da TGFe, é possível identificar quais pacientes necessitam de acompanhamento com o especialista e aqueles que podem ser mantidos em seguimento com a equipe da atenção primária.

## Avaliação Laboratorial da Função Renal

### Medida de Creatinina no Soro

A avaliação clínica da função renal faz parte dos cuidados médicos de rotina, sendo a creatinina sérica o exame laboratorial mais amplamente utilizado para avaliar a função renal. Em uma avaliação no Canadá, a creatinina foi o segundo exame laboratorial mais solicitado para pacientes ambulatoriais e o terceiro em internados e em emergência^3^.

Como a creatinina é predominantemente eliminada por filtração glomerular, com secreção de apenas uma fração mínima pelas células tubulares, a concentração sérica de creatinina apresenta associação inversa com a função renal. Uma elevação correspondente ao dobro da concentração sérica de creatinina significa redução aproximada de 50% da TFG.

A creatinina é o produto do metabolismo e degradação da creatina e da fosfocreatina nas células musculares esqueléticas, sendo gerada a uma taxa relativamente constante e, consequentemente, liberada de forma constante para a circulação sanguínea. Dado o seu baixo peso molecular, possui grande difusão através de todas as membranas celulares, estando presente no plasma sanguíneo, nos demais fluidos biológicos e no interior dos eritrócitos.

A produção de creatinina é dependente, em grande parte, da massa corporal magra e de algumas outras características do indivíduo, como idade, sexo, hábitos alimentares e atividade física. Em relação a essas características, destacam-se os extremos de idade (nas faixas pediátricas e geriátricas), a ingestão ou não de proteínas de origem animal (reduzida ou ausente em vegetarianos e veganos), e os extremos de massa muscular (desnutrição, sarcopenia). Merecem atenção casos especiais, como pessoas que tenham tido membros amputados ou em estados avançados de caquexia. A concentração de creatinina na circulação pode estar acima dos valores basais do indivíduo algumas horas após uma refeição com alto teor de proteínas de origem animal^4^.

A via de excreção da creatinina é, predominantemente, por filtração glomerular; mas, em condições de normalidade, cerca de 7% a 10% da creatinina urinária têm origem na secreção tubular^5^.

No sangue total, a creatinina é estável por 24 horas; no soro, a estabilidade é de até 7 dias à temperatura ambiente (20–25°C) ou refrigerada entre 2 e 8°C. Em amostras de soro congeladas a menos 20°C, é estável por até 3 meses, mesmo com ciclos de congelamento e descongelamento^6^.

Os métodos disponíveis para a mensuração da creatinina sérica e urinária incluem o colorimétrico baseado na reação de Jaffé e o enzimático, ambos potencialmente rastreáveis pelo método de espectrometria de massas e diluição isotópica (ID-MS, do inglês *Isothope Dillution Mass Spectrometry*), e passíveis de serem calibrados com Material de Referência de Padrões do Instituto Nacional de Padrões e Tecnologia (NIST SRM). Os ensaios enzimáticos são menos influenciados por cromógenos não creatinina em comparação com os colorimétricos^7,8^, apresentando menor variabilidade e, dessa forma, sendo mais indicados para a rotina laboratorial. Porém, são mais caros e não foram adaptados para todas as plataformas analíticas automatizadas disponíveis no mercado brasileiro^9,10^.

É referida como creatinina rastreável aquela mensurada por um conjunto diagnóstico cuja calibração seja rastreável a padrões de referência internacionais mensurados por ID-MS. A creatinina é determinada em material humano de referência pelo Instituto Nacional de Padrões e Tecnologia por ID-MS, que disponibiliza os padrões aos fabricantes de conjuntos diagnósticos para que realizem a calibração dos seus ensaios^11^.

Algumas substâncias endógenas, na dependência de sua concentração, podem interferir significativamente com os resultados obtidos pelo método baseado na reação de Jaffé: bilirrubina, glicose, proteínas, piruvato, ácido β-hidroxibutírico, proteínas, além de muitos fármacos, como cefalosporinas, dobutamina, lidocaína^12^. Amostras hemolisadas ou contendo hemoglobina fetal acima dos intervalos de referência podem fornecer resultados negativos de creatinina com a reação de Jaffé. Amostras lipêmicas podem fornecer resultados inexatos, independentemente do método utilizado^12^.

Como a urina, em geral, contém pouca ou nenhuma proteína em relação ao soro e não possui cromógenos, tanto o método enzimático quanto o de Jaffé são adequados para a medição da creatinina nessa matriz^13^.

É importante referir que a influência de substâncias interferentes é maior em concentrações de creatinina baixas, ou seja, dentro do intervalo deEstimated glomerular filtration referência, do que em concentrações mais elevadas, e que a padronização da calibração não minimiza as interferências analíticas^13^.

Em geral, as crianças apresentam concentrações de creatinina sérica mais baixas do que as dos adultos, possuindo, portanto, intervalos de referência próprios. Ainda que possa haver diferenças entre populações, os limites definidos pelo estudo *Canadian Laboratory Initiative in Pediatric Reference Intervals* (CALIPER)^13^ devem nortear os laboratórios e os pediatras na interpretação mais acurada dos resultados dos exames. Existem alguns estudos de transferência para outras plataformas analíticas e populações locais, conforme recomendado pelo CLSI^14,15^. Outra fonte de intervalos de referência para creatinina sérica em crianças é o banco de dados do *Pediatric Reference Range Initiatives da International Federation for Clinical Chemistry and Laboratory Medicine* (IFCC)^16^.

### Depuração de Creatinina

Não se recomenda considerar os limites de valores de referência da creatinina sérica como índice de avaliação da função renal, sendo a TFG estimada (TFGe) considerada mais sensível e específica como marcador da função renal^17^.

A TFG é mensurada pela depuração ou clearance de uma substância filtrada livremente pelos glomérulos, idealmente sem sofrer reabsorção ou secreção tubular e sem ser metabolizada ou eliminada por vias extrarrenais, sendo expressa em mL/min/1,73 m^2^. O cálculo da depuração é realizado pela fórmula (UV/P) × (1,73/A), sendo U = concentração urinária da substância; V = volume urinário, por unidade de tempo, em geral, por minuto; P = concentração plasmática da substância; e A = superfície corporal do paciente. A parte da fórmula (1,73/A) permite que o resultado seja normalizado para uma superfície corporal padrão de 1,73 m^2^. As concentrações sérica e urinária devem ser expressas nas mesmas unidades, o volume urinário expresso em mL e o tempo de coleta, em minutos, devem ser medidos com grande precisão^17^.

A TFG é medida com maior precisão pela utilização de substâncias exógenas, como inulina, alguns radioisótopos, tais como Cr^51^-ácido etilenodiamino tetra-acético (Cr^51^-EDTA), I^125^-iotalamato, Tc^99m^-ácido dietilenotriamino penta-acético (Tc^99m^-DTPA) ou meios de contraste radiográfico, como o iohexol. Esses procedimentos requerem que alguma substância seja injetada no paciente, são tecnicamente complexos, exigem equipamentos especiais, são demorados, de elevado custo e com possíveis efeitos colaterais. Por essas razões, na prática clínica, marcadores endógenos têm sido utilizados para medir ou estimar a TFG, com o intuito de investigar DRC, destacando-se a creatinina e a cistatina C séricas^18^.

O tempo de coleta da urina deve ser tal que permita uma avaliação com o menor grau de variações pontuais, sendo, habitualmente, de 24 horas ou 12 horas. Essas coletas prolongadas, no entanto, introduzem eventuais causas de erro, seja na medição do volume de urina, seja na marcação do tempo. Dessa forma, períodos mais curtos podem ser utilizados, visando à exatidão em cronometrar o tempo de coleta de urina e o esvaziamento completo da bexiga antes da primeira e da última coleta de urina. Outra condição crítica é o estado de hidratação do paciente durante a prova. O recomendado é que, ao iniciar a prova, o paciente receba uma carga hídrica de 20 mL/kg de peso corporal de água, seguindo com reposição equivalente ao volume urinário produzido em cada período. A utilidade diagnóstica da depuração de creatinina depende muito da adesão ao protocolo padronizado para coleta de amostras de sangue e urina^19^.

Quando a filtração glomerular está dentro dos intervalos de referência, a depuração de creatinina é praticamente superponível à da inulina. Resultados 7% a 10% mais elevados são observados pela secreção tubular, mas, à medida que se instala a insuficiência renal, a secreção tubular de creatinina se torna mais significativa, o que leva à superestimativa da TFG pela depuração de creatinina sérica. Adicionalmente, ocorre aumento da depuração extrarrenal da creatinina, com aumento de bactérias intestinais com atividade de creatininase^5^.

Algumas drogas atuam diretamente nas células tubulares renais, influenciando sua capacidade de secretar a creatinina, alterando, portanto, a proporção entre a creatinina filtrada e a secretada. Dentre as drogas que reduzem a secreção tubular são referidos os salicilatos, cimetidina, trimetoprima, quinina e procainamida, entre outras. Das drogas que aumentam a secreção tubular se relacionam triantereno, espironolactona e amilorida^5^.

A variação circadiana individual pode ser de até 25%, e os intervalos de referência, para indivíduos adultos são: homens, de 85 a 125 mL/min; e mulheres, de 75 a 115 mL/min. A TFG diminui, fisiologicamente, com o envelhecimento em cerca de 1 mL/min/ano de idade^20,21^. Na gravidez, por causa do considerável aumento do fluxo sanguíneo renal, a depuração da creatinina aumenta em 50% ou até mais^22^.

Outro conceito importante é a reserva funcional renal, caracterizada como a capacidade dos rins em aumentar a TFG em resposta a estímulos fisiológicos ou patológicos^23^. O estímulo conhecido e mais comum que estimula essa reserva, aumentando a TFG, é a sobrecarga de proteínas. A reserva funcional renal é calculada pela diferença entre a TFG estimulada (aumentada pelo estímulo) e a TFG basal. Alguns exemplos práticos são descritos a seguir: indivíduos vegetarianos têm TFG basal menor que a da população geral e maior reserva funcional renal; pacientes com nefropatias crônicas e rim único usualmente têm reserva funcional renal reduzida ou ausente, mesmo se tiverem TFG normal^23^.

Como principal medida da função renal utilizada na prática clínica atual, recomenda-se a medida da creatinina sérica e a estimativa da TFG. Valores de referência de normalidade para creatinina sérica não são recomendados para guiar a interpretação dos testes laboratoriais, devendo ser informada a TFGe em todo exame de creatinina sérica^5^.

### Medida de Cistatina C no Soro

Diferentemente da creatinina, a concentração sérica de cistatina C é menos dependente de idade, sexo, etnia, dieta e massa muscular^24,25^. Há referências a alterações na sua concentração em pacientes em uso de corticoides^26^, com processos neoplásicos^27^ e com doença tireoidiana^28^.

A cistatina C é uma proteína de baixo peso molecular produzida de forma contínua e constante por, praticamente, todas as células nucleadas do organismo humano, sendo removida da corrente sanguínea exclusivamente por filtração glomerular. Após a filtração, é reabsorvida e metabolizada sem sofrer secreção no túbulo proximal^29^. Consequentemente, apesar de a cistatina C ser livremente filtrada pelos glomérulos, sua depuração urinária não pode ser avaliada^29^.

A cistatina C pode ser medida no soro por imunoensaios, como método nefelométrico com intensificação da reação por partículas (PENIA) ou método imunoturbidimétrico com intensificação da reação por partículas (PETIA)^30^. Assim como a creatinina, a cistatina C também se tornou rastreável através do material de referência ERM-DA471/IFCC^31^, e as equações para a estimativa da TFG devem utilizar valores de cistatina C obtidos por metodologias validadas pelo IFCC^32^.

A medida de cistatina C, porém, é mais cara e nem sempre tão fácil e acessível em muitos laboratórios quanto a de creatinina, sendo mais reservada para confirmação de resultados ou nos casos em que a medida da creatinina possa sofrer interferências pré-analíticas. Os intervalos de referência descritos diferem pouco em relação a sexo e idade. Para as mulheres, é referido o intervalo de 0,52 a 0,90 mg/L, com média de 0,71 mg/L, e para os homens, de 0,56 a 0,98 mg/L com média de 0,77 mg/L^30^. À semelhança da creatinina, os valores diminuem a partir do nascimento até o primeiro ano de vida, permanecendo relativamente estáveis na vida adulta^33,34^.

A quantificação do seu nível sérico é mais sensível do que a da creatinina para a avaliação da função de filtração glomerular renal^35,36^, sendo um biomarcador melhor na detecção precoce do declínio da função renal, tanto em adultos quanto em crianças. As diretrizes do *National Institute for Health and Care Excellence* (NICE) do Reino Unido concluíram que a estimativa da TFG com a cistatina C é mais específica para prever desfechos importantes da doença do que o uso de creatinina^37^.

## Considerações Sobre as Equações para a Estimativa da Taxa de Filtração Glomerular

### Equações para Uso em Adultos

Os primeiros estudos realizados para a estimativa da função renal utilizando fórmulas com base na creatinina datam de meados do século XX^38,39^. Uma das equações mais conhecidas para a estimativa da TFG foi desenvolvida por Donald W. Cockcroft e M. Henry Gault, em 1976^40^. Os autores utilizaram dados de 249 homens com depuração de creatinina entre 30 e 130 mL/min. Foram considerados os parâmetros idade, sexo, peso corporal e concentração da creatinina. Essa equação não foi parametrizada para a superfície corporal padrão e não foi desenvolvida com creatinina padronizada pelo ID-MS. Além disso, a fórmula foi desenvolvida considerando como padrão de referência a depuração de creatinina na urina de 24h, sendo uma estimativa da depuração de creatinina e não precisamente da TFG^40^. Portanto, seu uso não é recomendado para finalidades clínicas, restringindo-se à pesquisa. A única vantagem atual dessa fórmula é que pode ser calculada manualmente, sem a necessidade de aplicativos.

Em 1999, o estudo Modificação da Dieta na Doença Renal (MDRD) apresentou uma equação^41^ desenvolvida a partir de uma coorte de 983 homens e 645 mulheres, adultos, predominantemente brancos, com diferentes níveis de doença renal, inscritos em uma pesquisa cujo objetivo era avaliar o efeito de dietas com baixo teor de proteína na progressão da DRC. Originalmente, essa fórmula incluía seis parâmetros: idade, sexo, raça, nitrogênio ureico, albumina e creatinina séricas, tendo sido posteriormente simplificada para apenas quatro variáveis: idade, sexo, raça e creatinina sérica, sendo identificada como 4-MDRD^42^. Em ambas as equações, há um fator específico com a finalidade de discriminar indivíduos da raça negra e um fator de ajuste para a superfície corporal padrão de 1,73 m^2^. Tendo em vista a imprecisão da estimativa em níveis de TFG elevados, o *National Kidney Disease Education Program* (NKDEP) recomenda que, quando a TFGe estiver acima de 60 mL/min/1,73 m^2^, o valor seja reportado apenas como superior a esse limite.

Uma segunda modificação dessa fórmula ocorreu em 2009, ao ser introduzida a medida da creatinina sérica usando ensaios com calibração rastreável aos materiais de referência internacionais aferidos por metodologia de espectrometria de massas e diluição de isotópica (MS-ID)^43^. Para a derivação dessa equação, foram utilizados os dados de 1.628 pacientes com DRC em diferentes graus, todos participantes do mesmo estudo MDRD. A TFG foi medida diretamente com o iotalamato-I^125^. Essa equação mostrou ter maior exatidão e precisão quando comparada com a equação 4-MDRD anterior, especialmente para TFG acima de 60 mL/min/1,73 m^2^. Foi mantido um coeficiente discriminatório diferenciado para pacientes de raça negra^43^. Essa equação tem sido amplamente utilizada, inclusive no Brasil, onde o critério de raça é menos marcante.

Em 2009, foi desenvolvida uma nova equação, CKD-EPI (*Chronic Kidney Disease – Epidemiology*), com maior acurácia que a MDRD para a estimativa da TFG^44^. Em 2022, o KDIGO (*Kidney Disease: Improving Global Outcomes*) desenvolveu nova versão de fórmula para a estimativa da TFG utilizando a creatinina sérica como biomarcador e excluindo qualquer fator de correção relativo à raça^45^. Essa equação foi desenvolvida a partir dos dados de duas metanálises, sendo que uma delas incluiu 10 estudos com 8254 participantes, com 31,5% negros, utilizando a medida da creatinina sérica e outra incluindo 13 estudos com 5352 participantes, sendo 39,7% de negros, utilizando a medida da creatinina sérica.

As Sociedades Científicas recomendam a substituição das equações anteriormente utilizadas por equações que não discriminam os pacientes pelo critério raça e utilizam apenas os valores de creatinina ou a associação de creatinina e cistatina C. Os estudos de validação já disponíveis demonstram que essas equações são suficientemente precisas e inclusivas^46^.

É razoável acreditar que a nova equação se mostre mais adequada em relação à população brasileira, uma vez que, em sua formulação, foi considerada a média das diferenças observadas entre os participantes de cada raça. O efeito imediato da aplicação da equação sem discriminação de raças não afetará, significativamente, os pacientes não negros, mas aumentará a chance de diagnóstico de DRC nas pessoas negras, possibilitando a instituição de tratamento mais precoce.

Vale lembrar que as novas equações, sem o critério de raça, também foram desenvolvidas a partir de dados obtidos de pacientes adultos, em atendimento ambulatorial, sem comorbidades significativas. Dessa forma, as limitações de uso descritas para as equações anteriores permanecem válidas.

Equações para adultos

Cockcroft e Gault (1976)

TFGe (mL/min) = [(140 – idade) × peso × (0,85 se for mulher)]/72 × creatinina.

4-MDRD

TFGe (mL/min/1,73 m^2^) = 175 × creatinina^–1,154^ × idade^–0,203^ × 1,212 (se negro) × 0,742 (se mulher).

CKD-EPI (2009)

TFGe (mL/min/1,73 m^2^) = 141 min (creatinina/k, 1)^α^ max (creatinina/k, 1)^–1,209^ × 0,993^idade^ × 1,018 (se mulher) × 1,159 (se negro).

k é 0,7 para mulheres e 0,9 para homens; α é 0,329 para mulheres e 0,411 para homens; min é a creatinina mínima dividida por k ou por 1; e max é a creatinina máxima dividida por k ou por 1.

CKD-EPI (2012) utilizando apenas cistatina C

eGFR (mL/min/1,73 m^2^) = 133 × min (cistatina/0,8, 1)^–0.499^ × max (cistatina/0.8, 1)^–1,328^ × 0,996^idade^ × 0,932 (se mulher).

CKD-EPI (2012) usando creatinina e cistatina

TFGe (mL/min/1,73 m^2^) = 135 min (creatinina/k, 1)^α^ × máx (creatinina/k, 1)^0,601^ × min (cistatina/0,8, 1)^0,375^ × máx (cistatina/0,8, 1)^0,711^ 0,995^Idade^ × 0,969 (se mulher) × (1,08 se preto)

k é 0,7 para mulheres e 0,9 para homens, α é 0,248 para mulheres e 0,207 para homens.

CKD-EPI (2021) usando apenas creatinina

TFGe (mL/min/1,73 m^2^) = 142 × min (creatinina/k, 1)^α^ × max (creatinina/k, 1)^1,200^ × 0,9938^Idade^ × 1,012 (se mulher).

k é 0,7 para mulheres e 0,9 para homens; α é 0,241 para mulheres e 0,302 para homens.

CKD-EPI 2021 usando creatinina e cistatina C

TFGe (mL/min/1,73 m^2^) = 135 × min (creatinina/κ, 1)α × max (creatinina/κ, 1)^−1,200^ × min (cistatina/0,8, 1)^−0,323^ × max (cistatina/0,8, 1)^–0,778^ × 0,9961^idade^ × 0,963 (se mulher).

k é 0,7 para mulheres e 0,9 para homens, α = −0,219 (para mulheres) e −0,144 (para homens), min é a creatinina mínima dividida por k ou por 1; e max é a creatinina máxima dividida por k ou por 1.

Obs.: em todas as equações, a idade é expressa em anos, a creatinina sérica em mg/dL, cistatina C em mg/L, o peso corporal em quilogramas.

### Equações para Uso em Pediatria

Atualmente, existem mais de 10 equações para estimar a TFG na população pediátrica, sendo recomendada a aplicação delas em crianças acima de dois anos de idade^2^. Da mesma forma que ocorre nas equações destinadas aos adultos, algumas delas se baseiam exclusivamente na creatinina sérica; outras, na cistatina C; e outras, ainda, na combinação desses dois biomarcadores.

Uma das equações para estimativa da TFG em crianças foi descrita por Schwartz e cols., em 1976, e relaciona uma constante, a altura corporal da criança e a concentração de creatinina sérica^47^. A partir da análise estatística dos dados de 186 crianças, os autores derivaram uma fórmula que permite uma estimativa da TFG, inclusive parametrizando para uma superfície corporal padrão de 1,73 m^2^. Ao ser validada em um grupo de 223 crianças, a equação se revelou bastante concordante com a depuração medida diretamente, seja pela depuração de creatinina, seja pela depuração de inulina.

No mesmo ano, Counahan e cols. publicaram outra equação, utilizando uma constante única e a relação da altura da criança com o valor da creatinina sérica^48^.

Em 1987, Schwartz e cols. publicaram uma modificação da equação original, na qual o valor da constante era relacionado à idade da criança. Os demais parâmetros foram mantidos^49^.

As equações consideradas como as mais adequadas, no momento, para estimar a TFG em crianças são as desenvolvidas e validadas pelo estudo Chronic Kidney Disease in Children (CKiD), publicadas em 2009 e 2012, denominadas de equações CKiD Schwartz, que relacionam creatinina isolada ou em associação com cistatina C séricas, nitrogênio ureico sanguíneo (BUN), altura corporal e sexo biológico da criança.

Elas foram desenvolvidas a partir dos dados de crianças com DRC, utilizando método enzimático de medição de creatinina, rastreável por espectrometria de massas por diluição de isótopos (IDMS). Essas equações se mostraram bastante precisas, especialmente na faixa entre 15 e 75 mL/min/1,73 m^2^. Segundo os autores, valores acima de 75 mL/min/1,73 m^2^ devem ser relatados apenas como tal e não como um número específico^50,51^.

Apesar da precisão dessas equações, há o inconveniente da necessidade do conhecimento dos valores das constantes, como, por exemplo, a altura corporal, nem sempre disponíveis no momento da aplicação das fórmulas. Devido a isso e às particularidades e limitações da medida da creatinina, cálculos da TFGe na população pediátrica através de equações utilizando a cistatina C foram desenvolvidos, destacando-se a fórmula de Schwartz^51^.

## Evidências Sobre o Uso da TFG Estimada a Partir da Creatinina Sérica

A equação CKD-EPI é a recomendada para a estimativa da TFG em adultos^43^. No desenvolvimento das equações, foram utilizados modelos de regressão, usando creatinina sérica e dados demográficos.

Condições clínicas que cursam com consumo muscular ou em pacientes amputados, o uso da TFGe a partir da creatinina é limitado. Outras situações não relacionadas à filtração glomerular que influenciam o nível sanguíneo da creatinina e, consequentemente, a TFGe são: 1. Sexo biológico: os homens, em geral, apresentam níveis mais elevados de creatinina do que as mulheres para um mesmo nível de filtração glomerular; 2. Etnia: os níveis de creatinina são mais elevados em indivíduos afro-caribenhos para o mesmo nível de TFGe; 3. Ingestão dietética recente: carne e peixe cozidos contêm creatina, a qual é rapidamente absorvida; 4. Medicamentos (efeito *in vivo*): por exemplo, a cimetidina e a trimetoprima bloqueiam a secreção tubular da creatinina. Tipicamente, 7–10% da excreção da creatinina é tubular, percentual que aumenta com a perda da filtração glomerular; 5. Depuração extrarrenal da creatinina: torna-se mais significativa em pacientes com DRC devido à degradação resultante do crescimento excessivo de bactérias no intestino delgado; 6. A concentração de creatinina sérica se mantém no intervalo de referência até que ocorra queda da TFG da ordem de 50% em não idosos e cerca de 70% em idosos; e 7. Utilização da TFGe na injúria renal aguda: TFG não diminui de forma linear com o aumento da creatinina sérica, limitando, assim, o seu uso no contexto clínico de queda aguda da filtração glomerular^52^.

Adicionalmente, alguns problemas analíticos devem ser observados na utilização da TFGe a partir da creatinina: 1. Especificidade: pseudocromógenos (por exemplo, cetonas, ácido ascórbico, piruvato, guanidina, cefalosporinas, proteínas) podem resultar em reações falso positivas quando se utiliza o método colorimétrico de Jaffé; 2. Medicamentos (efeito analítico): por exemplo, o metamizol causa falso aumento da creatinina quando se utiliza o método colorimétrico de Jaffé, enquanto a fenindiona e a dipirona determinam diminuição da creatinina quando usados alguns métodos enzimáticos; 3. Interferências espectrais, tais como icterícia, lipemia e hemólise, podem dar resultados falsos, aumentados ou reduzidos, de creatinina, dependendo das condições do ensaio; e 4. Variação metodológica: os resultados podem diferir entre os laboratórios^53^.

## Correção por Raça Nas Equações para Estimativa da TFG: Recomendação Atual

A equação CKD-EPI, que é recomendada para a estimativa da TFG, quando leva em consideração a raça, pode superestimar a TFG, levando ao subdiagnóstico de DRC. Tal constatação foi verificada na população de negros nos Estados Unidos e está possivelmente relacionada ao fato de que a raça é um conceito social que não se correlaciona, necessariamente, com as diferenças genotípicas ou metabólicas subjacentes entre as pessoas. Os parâmetros raciais no cálculo da TFGe não consideram as pessoas que se identificam como multirraciais, que se recusam a responder às perguntas sobre a sua raça ou nas quais a raça é definida indevidamente com base em fatores fenotípicos arbitrários, como o tom de pele, o tipo de cabelo ou, até mesmo, o status social. Além de inadequado para o cálculo da TFG, o parâmetro “raça” tem sido questionado em diversos algoritmos na prática clínica^53,54^. A própria existência de possíveis diferenças biológicas entre diferentes “raças humanas” é arbitrária, uma vez que remonta ao século 18, para justificar o expansionismo europeu e a suposta superioridade da raça branca^52^, o que não faz sentido na prática médica.

Dado que a TFGe é frequentemente usada na prática nefrológica, essa imprecisão, ao se incluir o fator “raça” na equação, pode afetar, particularmente em pacientes negros, a forma como os medicamentos são administrados ou mesmo proscritos, a rapidez com que os doentes são diagnosticados e referenciados para a consulta nefrológica para o tratamento da DRC, a elegibilidade para ensaios clínicos e o acesso às listas de espera para transplante renal.

Em 2021, foi proposta uma nova equação do CKD-EPI sem o uso da correção por raça^55^. Mesmo sem essa correção, as equações para estimativa da TFG, incluindo a CKD-EPI, foram desenvolvidas e validadas, em sua maioria, para a população norte-americana^55^, sendo a acurácia para outras populações, como a brasileira, ainda pouco estudada^56^.

## TFG Estimada a Partir da Cistatina C

A cistatina C é uma proteína plasmática de baixo peso molecular (12,8 kDa) livremente filtrada pelo glomérulo. Como é totalmente eliminada da circulação apenas por essa via, tem potencial para ser utilizada como marcador da TFG^56^. A cistatina C é um inibidor endógeno da cisteína protease sintetizado por todas as células nucleadas e apresenta alta correlação com a estimativa da TFG. A sua produção é relativamente independente do sexo, massa muscular, influências dietéticas e período nictemeral, podendo ser influenciada por inflamação, processo neoplásico, disfunção da tireoide e tabagismo^57^.

Também foram desenvolvidas equações de estimativa da TFG baseadas na cistatina C^58^, incluindo equações que incorporam tanto a cistatina C quanto a creatinina^59^. Embora essas equações mostrem apenas melhorias modestas na acurácia da TFGe em comparação com as equações baseadas na creatinina, há, cada vez mais, evidências de que a utilização da cistatina C supera a creatinina na estratificação do risco, particularmente do risco cardiovascular. Adicionalmente, a TFGe baseada na cistatina C é menos influenciada pela idade, ingestão alimentar e raça, e pode ser benéfica em situações nas quais a relação entre a massa muscular, a creatinina e a TFG está comprometida (por exemplo, doenças consumptivas e em pacientes amputados).

As Diretrizes do KDIGO^2^ apresentam recomendações baseadas em evidências para determinação da TFGe, como descrito a seguir:

- Recomenda-se o uso da creatinina sérica ou sua depuração para a obtenção da TFGe para a investigação inicial (1A);
- Sugere-se o uso adicional de exames laboratoriais, tais como cistatina C ou sua estimativa da TFG como testes confirmatórios em circunstâncias específicas, quando a TFG baseada na creatinina sérica for menos fidedigna (2B);
- Recomenda-se que os clínicos usem a equação de estimativa da TFG derivada da creatinina sérica (TFGe) no lugar da creatinina sérica isolada (1B).

## Calculadoras On-line

Existem numerosas calculadoras disponíveis on-line para a resolução das equações que estimam a TFG, o que facilita, em muito, sua aplicação na rotina tanto dos clínicos quanto dos especialistas. Igualmente, com a introdução de sistemas informatizados nos laboratórios clínicos, tornou-se comum que a estimativa seja fornecida como um complemento à solicitação da mensuração de creatinina sérica. Evidentemente, essa prática ainda está consolidando-se em nosso meio e se restringe às equações que utilizam, unicamente, a creatinina. A introdução de equações baseadas na cistatina C isolada ou em associação com creatinina trará uma dificuldade operacional, uma vez que a medida desses dois parâmetros deverá ser explicitada pelo médico.

Abaixo, relacionamos alguns sites provedores de calculadoras.

https://sbn.org.br/medicos/utilidades/calculadoras-nefrologicas/ckdepi/

https://www.kidney.org/professionals/Kdoqi/gfr_calculatorPed

https://www.mdcalc.com/calc/43/creatinine-clearance-cockcroft-gault-equation

https://www.mdcalc.com/calc/3939/ckd-epi-equations-glomerular-filtration-rate-gfr

https://www.mdcalc.com/calc/10008/revised-schwartz-equation-glomerular-filtration-rate-gfr-2009

## Recomendações para os Laudos

As recomendações atuais em relação aos laudos dos exames laboratoriais são que:

- A concentração da creatinina sérica seja relatada em mg/dL e seu valor arredondado para o centésimo mais próximo de um número inteiro, o que equivale a dizer que deve ser um número com duas casas decimais.
- A estimativa da TFG seja incorporada ao laudo do resultado de creatinina e seu valor seja relatado e arredondado para o número inteiro mais próximo e relacionado à área de superfície corporal de 1,73 m^2^ em adultos, usando as unidades mL/min/1,73 m^2^.
- O valor de TFGe baseado na creatinina inferior a 60 mL/min/1,73 m^2^ seja relatado como “diminuído”.
- Se deve relatar o valor da concentração de cistatina C sérica arredondado para o centésimo mais próximo de um número inteiro quando expresso em unidades convencionais (mg/L).
- Se deve relatar os valores de TFGe baseados em equações que utilizem cistatina C isoladamente ou em associação com creatinina arredondados para o número inteiro mais próximo e em relação à área de superfície corporal, utilizando as unidades mL/min/1,73 m^2^.
- Os níveis de TGFe baseados em equações que utilizem cistatina C isoladamente ou em associação com creatinina inferiores a 60 mL/min/1,73 m^2^ sejam relatados como “diminuídos”.
- As equações utilizadas sejam claramente reportadas, incluindo-se suas referências bibliográficas.

## Situações em que não se Recomenda o Uso da TFG Estimada

As estimativas da TFG foram desenvolvidas em situações de estabilidade da creatinina sérica. Sabe-se que seu nível sérico é dependente de vários fatores, tais como massa e atividade muscular, dieta, secreção tubular e eliminação pelo trato gastrointestinal, fatores esses que não são considerados determinantes da filtração glomerular.

Em situações de instabilidade da concentração da creatinina, como lesão renal aguda, os erros de estimativa da TFG são maiores.

Para uso e interpretação adequados da TFGe, é necessária sua avaliação no contexto clínico do paciente. Extremos de idade, como crianças e idosos, têm particularidades que devem ser consideradas.

## Uso da TFG Como Ferramenta de Prevenção/Detecção Precoce da DRC

Os estudos relacionados ao diagnóstico precoce de DRC indicam que os exames laboratoriais que devem constar das avaliações iniciais são a pesquisa de proteinúria, ressaltando-se que o KDIGO recomenda a determinação da relação albumina/creatinina em amostra isolada de urina, e a determinação da concentração da creatinina sérica, sensibilizada pela determinação associada da estimativa da TFG. A estimativa permite diagnóstico precoce de maior número de casos de perda de função renal.

A triagem da DRC através da TFGe e da dosagem de albuminúria vem sendo recomendada para todos os pacientes com fatores de risco para DRC, como os pacientes hipertensos e diabéticos, por exemplo. Indivíduos com fatores de risco devem ser avaliados anualmente, podendo se orientar a reavaliação dos parâmetros TFGe e relação albumina/creatinina urinárias (RAC) semestralmente, conforme contexto clínico e resultados ou sua variação ao longo do monitoramento. Atualmente, recomenda-se a pesquisa de albuminúria por meio do exame em amostra isolada de urina (relação albumina/creatinina urinárias).

Pacientes com DRC estágio G4 e G5 devem ser referenciados para o nefrologista para seguimento e adequado manejo das complicações da doença^44^.

## Conclusão

Embora ainda insuficientemente utilizados, os níveis de proteinúria ou de albuminúria e a estimativa da TFG são recursos de grande valor na detecção e estadiamento da DRC, assim como na previsão do risco de progressão da DRC e de DCV. Ressaltamos que, no presente texto, focamos apenas na avaliação da TFG, sem desconsiderar que proteinúria e albuminúria são marcadores de lesão renal sensíveis e muito relevantes.

Seguindo as orientações da American Association for Clinical Chemistry (AACC) e da National Kidney Foundation (NKF), a Sociedade Brasileira de Patologia Clínica/Medicina Laboratorial (SBPC/ML) e a Sociedade Brasileira de Nefrologia (SBN) recomendam, para indivíduos adultos, o uso preferencial das seguintes equações para a estimativa da TFG:

- CKD-EPI (2021), apenas com creatinina sérica;
- CKD-EPI (2021), com creatinina e cistatina séricas e
- CKD-EPI (2012), apenas com cistatina C sérica.

Para a população pediátrica, as equações recomendadas pela NKF, SBN e SBPC/ML são:

- Equação de Schwartz (2009), baseada na creatinina sérica;
- Equação de Schwartz CKiD (2012), baseada na cistatina C sérica; e
- Equação de Schwartz CKiD (2012), baseada na creatinina e na cistatina C séricas.
