# Supplementary material for: Erratum: Estimated glomerular filtration rate in clinical practice: consensus positioning of the Brazilian Society of Nephrology (SBN) and Brazilian Society of Clinical Pathology and Laboratory Medicine (SBPC/ML)
Source: J Bras Nefrol. 2025 Apr 14;47(2):e20230193er. doi: 10.1590/2175-8239-JBN-2023-0193eren (PMC11996086; doi:10.1590/2175-8239-JBN-2023-0193eren)
Supplement: Translation

# Translation: Errata: Estimativa da taxa de filtração glomerular na prática clínica: posicionamento consensual da Sociedade Brasileira de Nefrologia (SBN) e Sociedade Brasileira de Patologia Clínica e Medicina Laboratorial (SBPC/ML)

No artigo “Estimativa da taxa de filtração glomerular na prática clínica: posicionamento consensual da Sociedade Brasileira de Nefrologia (SBN) e Sociedade Brasileira de Patologia Clínica e Medicina Laboratorial (SBPC/ML)”, com número de DOI: https://doi.org/10.1590/2175-8239-JBN-2023-0193pt, publicado no periódico Brazilian Journal of Nephrology, 46(3):e20230193, 2024, na página 6:

**Onde se lia:**

CKD-EPI (2021) usando apenas creatinina

TFGe (mL/min/1,73 m^2^) = 142 × min (creatinina/k, 1)^α^ × max (creatinina/k, 1)^1,200^ × 0,9938^Idade^ × 1,012 (se mulher).

k é 0,7 para mulheres e 0,9 para homens; α é 0,241 para mulheres e 0,302 para homens.

**Leia-se:**

CKD-EPI (2021) usando apenas creatinina

TFGe (mL/min/1,73 m^2^) = 142 × min (creatinina/k, 1)^α^ × max (creatinina/k, 1)^-1,200^ × 0,9938^Idade^ × 1,012 (se mulher).

k é 0,7 para mulheres e 0,9 para homens; α é 0,241 para mulheres e 0,302 para homens.
